# Lumbopelvic Muscle Mechanical Properties in Women with Multiple Sclerosis: A Cross-Sectional Case-Controlled Study

**DOI:** 10.3390/biomedicines12122925

**Published:** 2024-12-23

**Authors:** Inés Cruz-Medel, María Ángeles Peña-Toledo, Francisco Alburquerque-Sendín, Daiana Priscila Rodrigues-de-Souza, Cristina Conde-Gavilán, Ana María Jover-Sánchez, Claudia Carmona-Medialdea, Eduardo Agüera-Morales

**Affiliations:** 1 Dementia and Multiple Sclerosis Unit, Neurology Service, Reina Sofia University Hospital, 14004 Cordoba, Spain; angelespe87@gmail.com (M.Á.P.-T.); cristinaconde84@gmail.com (C.C.-G.); amjs@hotmail.es (A.M.J.-S.); claudiacm88@hotmail.es (C.C.-M.); doctoredu@gmail.com (E.A.-M.); 2 Department of Nursing, Pharmacology and Physical Therapy, University of Cordoba, 14004 Cordoba, Spain; falburquerque@uco.es (F.A.-S.); drodrigues@uco.es (D.P.R.-d.-S.); 3 Maimonides Institute for Biomedical Research of Cordoba (IMIBIC), Reina Sofia University Hospital, University of Cordoba, 14004 Cordoba, Spain

**Keywords:** lumbar paravertebral muscles, MyotonPRO device, pelvic floor muscles, multiple sclerosis

## Abstract

**Background/Objectives**: To compare the lumbopelvic muscle mechanical properties (MMPs) of women with and without multiple sclerosis (MS) and explore relationships between these properties and sociodemographic/clinical characteristics. **Methods**: This cross-sectional observational study included 22 women with MS and 22 age- and BMI-matched women without MS. MMPs (frequency, stiffness, decrement, relaxation, and creep) of pelvic floor and lumbar paravertebral muscles were assessed using a MyotonPRO device. Sociodemographic and clinical data related to pelvic floor health were also collected. **Results**: Women with MS showed significant differences in pelvic floor MMPs, including higher frequency (3.26 Hz; 95% CI [2.12, 4.41]), stiffness (90 N/m; 95% CI [55.09, 124.91]), and decrement (0.2; 95% CI [0.09, 0.31]), and lower relaxation (6.15 ms; 95% CI [8.26, 4.05]) and creep (0.24; 95% CI [0.34, 0.13]) compared to women without MS. For lumbar paravertebral muscles, differences were observed only on the right side, with lower frequency (2.15 Hz; 95% CI [0.28, 4.02]) and stiffness (62.17 N/m; 95% CI [10.7, 113.65]) in women with MS. Correlation patterns between MMPs and clinical characteristics differed by group, with moderate correlations found only in the MS group (e.g., EDSS: r = 0.57; *p* = 0.006; PFDI-20: r = 0.47; *p* = 0.026). **Conclusions**: Women with MS exhibit altered pelvic floor MMPs, characterized by reduced tone and stiffness and increased elasticity and viscoelasticity, while lumbar paravertebral differences are minimal. These findings highlight the need for objective MMP assessments in women with MS to guide preventive and therapeutic interventions.

## 1. Introduction

Multiple sclerosis (MS) is a chronic inflammatory disease that affects the central nervous system (CNS) [1] and causes demyelination, which is diagnosed in young adults (aged 20–40 years), predominantly in female patients [2,3]. MS causes alteration of the function of the pelvic floor (PF) which, although common in women [4], is aggravated by deterioration of the motor innervation of the external anal sphincter [5]. In fact, up to 80% of patients with MS are affected by lower urinary tract symptoms, manifesting as problems with urine storage, evacuation, or both [6]. Although these symptoms are not life-threatening, they have a significant negative impact on the quality of life of those affected [7].

The PF muscles (PFMs) play an important role in maintaining continence [8] and in lumbopelvic stability because increased PF tension stabilizes the sacroiliac joint, positions the sacrum, and improves the ability of the lumbar spine and pelvis to withstand mechanical stress resulting from increased pressure [9]. Furthermore, studies suggest that the correct configuration of the spine and its curvatures protect the pelvis and PF from direct intra-abdominal pressures, allowing for more efficient contraction of the PFMs [10,11]. This can be explained by the regional interdependence of these structures and allows us to describe existing clinical observations between different regions of the body [12]. However, to date, there is no specific knowledge available regarding the behavior of lumbopelvic muscle mechanical properties (MMPs) in MS, nor about their relationships with factors such as sociodemographic characteristics, type and degree of incontinence, or quality of life.

In this context, myotonometry represents a novel and non-invasive method to characterize MMPs. In this approach, a brief superficial mechanical impulse is applied to record different oscillation parameters of the muscle response in terms of tone, stiffness, elasticity, relaxation time, and fluence [13]. This technology is now being applied in different research fields including physiotherapy, oncology, rheumatology, neurology, and musculoskeletal disorders, among others [14]. From among the studies published using myotonometry on the musculoskeletal system, much of the work to date has focused on the paravertebral muscles of the lumbar spine [15], with good to excellent absolute and relative reliability results. Indeed, the intrarater and interrater reliability of PFMs with this device is good to very good in healthy women and in those with urinary incontinence (UI), thereby supporting the use of this device both in women with and without UI [16]. Furthermore, myotonometry has been shown to be clinically applicable, valid, and sufficiently reliable for measuring tone and other properties such as stiffness according to a phantom tissue model [17]. Finally, recent studies using myotonometry showed changes in muscle tone and elasticity of the superior orbitalis muscle in patients with MS when compared to healthy ones [18].

Thus, the primary aim of this study was to examine whether the lumbopelvic MMPs of women with MS differ from those of healthy controls. As a secondary objective, we aimed to identify possible relationships between the status of the MMPs and various sociodemographic and clinical characteristics in each group of women. Specifically, we aimed to assess muscle tone, stiffness, and elasticity of the PFM and lumbar muscles in women with MS and compare them to the corresponding values in healthy women. Investigating these MMPs is crucial for understanding how neurological impairment in MS affects muscle function, particularly in relation to common MS-associated dysfunctions such as incontinence and sexual dysfunction. By identifying specific alterations in muscle properties, we aim to provide valuable insights that can guide more targeted and personalized therapeutic interventions, ultimately improving the treatment strategies and quality of life for MS patients.

We hypothesize that women with MS exhibit altered mechanical properties in both the PFMs and lumbar muscles, including reduced strength, elasticity, and coordination, due to the neurological damage associated with MS. These alterations may contribute to an increased prevalence of pelvic floor dysfunctions, such as urinary and fecal incontinence, when compared to healthy women.

## 2. Materials and Methods

### 2.1. Design

This was a cross-sectional observational study of cases (women with MS with PF involvement) and controls (healthy women), carried out at the Reina Sofía University Hospital in the province of Cordoba (Spain). Before starting the work, the patients were informed about the procedure, duration, and objective of the study and signed the corresponding written informed consent document.

### 2.2. Participants

Women from the specialized MS clinic who met the following inclusion criteria were recruited through a non-probabilistic sampling of consecutive cases: (1) female patients of legal age (2) with MS diagnosed according to McDonald’s criteria (2017) [19], (3) with an Expanded Disability Status Scale (EDSS) score between 0.0 and 6.5, (4) receiving a disease-modifying treatment (DMT), (5) whose pharmacological treatment dose for spasticity had been stable in the 30 days prior, (6) who had undergone a previous neurological evaluation that had determined the EDSS score and defined its associated symptoms, and (7) were able to understand the objective of the work, complete the study procedures, and sign the informed consent document. The exclusion criteria were (1) being in a disease flare-up phase or having had a flare-up in the 30 days prior to the start of the study, (2) participating in another clinical trial with a drug or intervention, (3) being in the first three days of menstruation or pregnant, (4) a body mass index (BMI) exceeding 40 kg/m^2^, (5) carrying a metallic device such as a pacemaker or intrauterine device (IUD) that could interfere with the MyotonPRO result, (6) consumption of any substance that alters muscle tone, (7) patients with myopathies or concomitant peripheral nervous system diseases, and (8) an inability to collaborate or any other situation in which the evaluation could be altered. The control group also comprised women who met both the inclusion and exclusion criteria, except they did not have a diagnosis of MS. In addition, one-to-one matching was performed for each case according to age (±3 years) and BMI (±3 kg/m^2^), also considering whether the participants had given birth.

### 2.3. Data Collection

After providing informed consent, participants completed a registration form (age, BMI, vaginal deliveries) and the following validated questionnaires: the Spanish version of the Pelvic Floor Distress Inventory (PFDI-20) [20], assessing dysfunction across genital prolapse (6 items), colorectal–anal (8 items), and urinary (6 items) domains, with scores ranging from 0 to 300; the Spanish version of the Pelvic Floor Impact Questionnaire–Short Form (PFIQ-7) [20], evaluating the impact of urinary, colorectal–anal, and genital prolapse symptoms on daily activities, with scores from 0 to 300; the Spanish version of the Global Physical Activity Questionnaire (GPAQ) [21], measuring activity intensity, frequency, and duration; and a Visual Analogue Scale (VAS), scoring pain intensity from 0 to 10.

At the end of data collection, the PF and lumbar paravertebral musculature of the participants was evaluated. A MyotonPRO (Myoton AS, Tallinn, Estonia) [22,23], a small, non-invasive, and portable handheld device used to measure the MMPs, was used in this study. MyotonPRO was used to assess superficial musculature because it is designed to evaluate biomechanical properties through non-invasive mechanical vibrations, which only penetrate tissues close to the surface. This makes it ideal for measuring muscles accessible via the skin, as its technology does not reach deeper layers, providing precise data for clinical and research applications related to muscle monitoring and rehabilitation. The measurement consists of 3 main components: mechanical impulse stress, co-oscillation recording, and parameter calculation. The tip of the probe is 3 mm in diameter and is applied perpendicular to the skin surface, above the muscle being measured. A constant pressure (0.18 N) is applied, so that the superficial subcutaneous tissues are slightly compressed. Next, a brief (15 ms) low-force (0.4 N) mechanical impulse is transmitted to the underlying muscle. The muscle’s damped oscillation, recorded via accelerometer, quantifies key properties: tone, reflecting intrinsic tension at rest (Hz); biomechanical properties, including stiffness (N/m), resistance to deformation, decrement (logarithmic damped oscillation decay, inverse of elasticity), and recovery ability; and viscoelastic properties, such as stress relaxation time (ms), the time to recover shape post-deformation, and creep (Deborah number), representing gradual elongation under sustained tensile stress [24].

This allows for precise quantification of muscle characteristics such as tone, stiffness, elasticity, and relaxation time. In research, the MyotonPRO is invaluable for assessing muscle function in various clinical populations, including those with neurological conditions like multiple sclerosis. It offers objective, reproducible, and real-time measurements, making it an essential tool for evaluating subtle changes in muscle properties over time. By providing detailed, quantitative data, the MyotonPRO contributes to a deeper understanding of muscle impairments, facilitates early detection of dysfunction, and supports the development of targeted therapeutic interventions.

**Figure 1:**
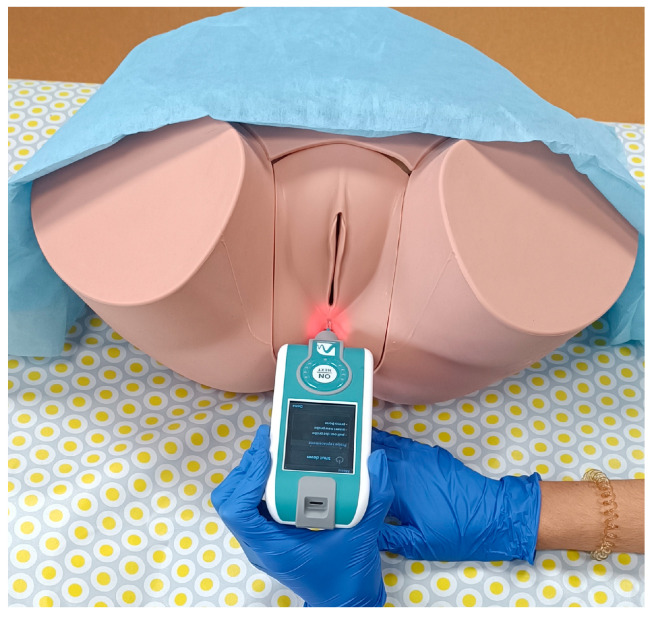
Measurement of the MMPs in the right area of the PF.

The PF MMP evaluation (Figure 1) was subsequently carried out, as described in detail and supported by different study protocols elsewhere [18,25]. For this, the volunteers were placed in a supine position with modified lithotomy, and a single measurement was taken on both the right and left sides of the central perineal core. The device was also used to measure lumbar muscle tone [26], where the volunteers were placed in a prone position, and a single measurement was taken on both the right and left sides of the lumbar paravertebral area at the L5-S1 level.

### 2.4. Sample Size

To ensure adequate statistical power for detecting meaningful differences in muscle stiffness between groups, we conducted a sample size calculation using the G*Power software (version 3.1.9.2). We aimed to identify a large effect size, represented by a Cohen’s d index of 0.87, which was calculated based on a minimum detectable change of 41.48 N/m and a pooled standard deviation of 47.95 N/m for muscle stiffness [18]. This large effect size was chosen to reflect clinically relevant differences in muscle stiffness measurements.

To achieve a Type I error rate (α) of 0.05 and a statistical power (1-β) of 0.80, the software determined that a sample size of 22 participants per group was required to detect significant differences using a two-tailed *t*-test for independent groups. These parameters ensure that the study is appropriately powered to minimize the risks of both Type I (false positive) and Type II (false negative) errors. The decision to select a large effect size was based on prior literature demonstrating that variations in muscle stiffness of this magnitude are clinically significant and detectable under controlled conditions. This effect size also aligns with the precision and reliability of the measurement tools used in our study. The pooled standard deviation of 47.95 N/m reflects variability in muscle stiffness measurements from previous studies and serves as a robust estimate for calculating the required sample size. This rigorous approach to sample size estimation ensures that our study is adequately powered to detect meaningful differences in muscle stiffness, thereby supporting the validity and reliability of our findings.

### 2.5. Statistical Analysis

Frequencies and percentages were used to describe the qualitative data as they provide an effective summary of categorical variables. The Kolmogorov–Smirnov test was applied to assess normality, and since MMPs and BMI variables showed a normal distribution (test *p* > 0.05), they were described by means, standard deviations, or 95% confidence intervals (95% CI). Age and questionnaire variables were not normally distributed and were described by their medians and interquartile ranges.

To assess the between-group comparability, sociodemographic and clinical data were compared with unrepeated Student *t*-tests and Mann–Whitney U tests for non-normally distributed data. Fisher and χ^2^ tests were used for the qualitative data. For the study’s primary aim, unpaired Student *t*-tests were applied to identify differences in the MMPs of the PF and lumbar muscles between groups.

To identify intra-group associations between the MMPs and sociodemographic and clinical features, Pearson *r* and Spearman *p* (*r*s) coefficients were calculated for normally distributed data and for non-normally distributed or ordinal data, respectively. Correlations were considered negligible (0.0 to 0.19), fair (0.20 to 0.39), moderate (0.40 to 0.69), strong (0.70 to 0.89), or almost perfect (0.0 to 1.00) [27].

All the comparisons were bilateral, and probabilities exceeding 95% (alpha *p*-values less than 0.05) were considered significant. The statistical analysis was performed using SPSS software (version 28, IBM Corp., Armonk, NY, USA).

## 3. Results

### 3.1. Sociodemographic and Clinical Variables

For this study, 22 women with MS were recruited and formed the case group, while 22 nulliparous women without MS formed the control group. There were no differences in the mean age of the women with MS compared to those in the control group. In reference to the number of births, 45.45% of patients with MS were nulliparous compared to 50% in control group, with no significant differences between the two. In turn, the average score on the PFDI-20 and PFIQ-7 questionnaires was notably higher in the patient cases than in the controls. For the GPAQ questionnaire, the cases indicated that the patients engaged in less intense physical activity than the controls, with statistically significant differences. Regarding UI, as set out in Table 1, 36.36% of the patients with MS presented urgency UI (UUI) and 31.82% of the controls reported stress UI (SUI). Finally, in women with MS, the average time from diagnosis of the disease had been approximately 10.77 years; their average score on the EDSS scale was 2.89 points; 100% were on a DMT; and a high percentage had not received treatment for spasticity, nor neurorehabilitation or botulinum toxin treatment. None of the women indicated the presence of any pain during the evaluations (VAS = 0).

### 3.2. Comparisons of Lumbopelvic Muscle Mechanical Properties Between the Groups

Significant differences between the cases and controls were identified for all the MMPs of the PF. We found differences on the right side of the PF, with some of the variables being higher in women without MS, with a frequency of 3.26 Hz (95% CI [2.12, 4.41]), stiffness of 90 N/m (95% CI [55.09, 124.91]), and decrement of 0.2 (95% CI [0.09, 0.31]), compared to patients with MS. Likewise, the relaxation and creep values were lower in the controls by 6.15 ms (95% CI [8.26, 4.05]) and 0.24 ms (95% CI [0.34, 0.13]) compared to the cases. There were also significant differences in the variables for the left side of the central nucleus of the perineal muscle between women with and without MS, with the cases presenting a frequency of 2.82 Hz (95% CI [1.53, 4.12]), stiffness of 77.5 N/m (95% CI [36.27, 118.73]), and decrement of 0.19 (95% CI [0.07, 0.31]) more than women without the disease. The relaxation and creep variables were also significantly different between women with and without MS, with women without MS showing values of 5.55 ms (95% CI [7.9, 3.21]) and 0.21 (95% CI [0.3, 0.11]) lower than those with MS. Thus, it is worth highlighting that the frequency, stiffness, and decrement variables trended to be higher, and the relaxation and creep variables trended to be lower, in women without MS compared to those with the disease (Table 2). These findings suggest that women with MS have altered pelvic floor muscle function, which may contribute to symptoms like incontinence. In this way, a proper intervention could help improve muscle function, reduce spasticity, and enhance pelvic floor health in MS patients.

Fewer statistically significant differences were identified between the groups for the MMPs of the lumbar paravertebral muscles. Only the *frequency* and *stiffness* variables for the right area differed between women with and without MS. At 2.15 Hz (95% CI [0.28, 4.02]), the right *frequency* values were higher in women without MS compared to women with MS. Higher values were also evident for right-side *stiffness* in healthy women compared to those with MS, at 62.17 N/m (95% CI [10.7, 113.65]). These results are shown in Table 3.

### 3.3. Intragroup Relationships Between the Lumbopelvic Muscle Mechanical Properties and Sociodemographic and Clinical Data

Very few correlations were identified for the lumbopelvic MMPs, with different patterns in each group. Women with MS only presented a moderate positive correlation between the PF MMPs and the PFDI-20 questionnaire and EDSS scale results for relaxation and creep on the right side (EDSS: r = 0.50; *p* = 0.017 and r = 0.57; *p* = 0.006, respectively) and a moderate positive correlation on the left side for stiffness and decrement (PFDI-20: r = 0.43; *p* = 0.049 and r = 0.47; *p* = 0.026, respectively). Regarding the correlations between the PMMs of the lumbar paravertebral muscles, a moderate positive correlation has been shown for the variables frequency and stiffness of the left side with age (r = 0.45; *p* = 0.042 and r = 0.47; *p* = 0.033, respectively). However, there were no correlations between the MMPs of the lumbar paravertebral muscles and the clinical variables, the EDSS scale, or the questionnaires.

In women without MS, no correlations were identified between the MMPs of the PF and the sociodemographic and clinical variables or with the questionnaires. In this population group, the MMPs of the lumbar paravertebral muscles were only moderately correlated with age and the number of births. Age was positively related to the right zone for frequency, stiffness, decrement, relaxation, and creep, and with relaxation and decrement in the left zone. Regarding the number of births, a moderate interaction with the lumbar MMPs of the left zone was observed for frequency (r = 0.57; *p* = 0.006), stiffness (r = 0.51; *p* = 0.015), relaxation (r = 0.54; *p* = 0.009), and creep (r = 0.54; *p* = 0.01).

## 4. Discussion

This present study identified differences in the MMPs of the PF between women with and without MS, meaning that this disease could influence the MMPs of the lumbopelvic region. Furthermore, there were some specific differences in the MMPs of the lumbar paravertebral muscles in healthy women compared to women with MS. It was also possible to identify different correlation patterns between MMPs and clinical characteristics, depending on whether the participant had MS or not. Finally, it is worth noting that none of the women withdrew from the study or reported pain or discomfort as a result of the study evaluation techniques.

Compared to women without MS, there was less muscle tone and stiffness on both sides of the central nucleus of the perineal area in the women with MS, with the differences found in this present study being slightly higher than these values (a minimal detectable change (MDC) of less than 2 Hz and 52.34 N/m [18]). Furthermore, the elasticity and creep of both sides was greater in healthy women compared to those with MS. In this sense, there is evidence suggesting that more than a third of patients with MS experience signs of PF weakness [28], along with preservation of peripheral muscle function caused by CNS lesions [5] and the presence of plasticity in antigravity muscles [29] such as the triceps, quadriceps femoris, gluteus medius, and hip adductor, among others [30]. The spinal cord is a key part of the CNS and is the main source of neurogenic dysfunction of the lower urinary tract, which occurs in 75–80% of patients with MS [30,31] (even reaching almost 100% 10 years after diagnosis of the disease [32]) and which correlates with increased EDSS scores [33]. Furthermore, MMP involvement has previously been described for other PF dysfunctions such as UI [7], although in this present work we identified an increase in PF tone and stiffness. Variations in MMPs, such as contractility, elasticity, strength, and fatigue, in women with MS may indicate neuromuscular dysfunction and affect mobility. These changes suggest the need for specific interventions, such as strength and flexibility exercises, techniques to reduce spasticity, and tailored resistance training programs to address muscular fatigue. Identifying and addressing these variations in treatment is essential to improve muscle function, postural stability, and quality of life in patients with MS, optimizing their mobility and minimizing the progression of muscular dysfunction [34].

Regarding the MMPs of the lumbar paravertebral muscles, the tone and stiffness of the right side was lower in women who had MS compared to those who did not. This may be because of the fact that patients with MS tend be less physically active than healthy people [35] and are therefore more oriented towards greater resistance and muscle strengthening [36]. Furthermore, 97% of patients with MS present spinal cord involvement, with 55% reporting low back pain and gait disorders as possible causes of physical inactivity [37].

In relation to clinical characteristics, there was evidence of a moderate relationship between the MMPs of the PF of the right zone and the EDSS score in women with MS, probably partly because the gut/bladder system is one of the seven functional systems used to calculate the EDSS score, and so this assessment also includes symptoms such as UI. In turn, the tone and speed of recovery of the PF tissue of the left zone in women with MS has also previously been related to the results of the PFDI-20 questionnaire because the state of the PF MMPs is used as a reference to assess the clinical status of this musculature [18]. However, it is important to note that these correlations do not necessarily imply causality.

A positive relationship was also identified between age and lumbar MMPs in both women with and without MS because lumbar spine degeneration caused by biomechanical stress and the consequent effects this has on the paravertebral musculature is a general artefact of the aging process [37]. This muscle involvement has been evidenced as an increased degree of trunk flexor and extensor muscle co-contraction [38], which thereby results in the previously described increased tension and stiffness. Furthermore, age-related degenerative disorders of the lumbar spine have not been previously studied in patients with MS, while MS lesions may be misdiagnosed as lumbar spine disease [37]. Finally, in healthy women, there is a positive correlation between the tone of the PF in the left zone and the number of births. This asymmetry of behavior between the left and right sides could be related with the presence, in up to 80% of healthy individuals, of muscle fascial tension, with multiparous women presenting a greater tendency towards asymmetries that, in turn, alter PFM contraction and biomechanics of the spine and lower extremities [25]. Musculoskeletal dysfunction of these muscles also results in a loss of support from the PFMs, which contributes to UI in 46% of cases [33].

Finally, the strengths of this study lie in the fact that no previous work published in the scientific literature has described lumbopelvic MMPs in patients with MS, making our study pioneering in the evaluation of these variables in this patient population. The main limitation of this study was the sample size in both population groups, which may affect the statistical power and generalizability of the findings, as well as the heterogeneity in the progression of MS observed across participants. Additionally, the subjectivity of the questionnaires used, which depend on the interpretation and responses provided by the patients, introduces variability based on individual perceptions and understanding, potentially influencing the consistency and accuracy of the results. Another limitation is the use of a single measurement of the MMPs, which may not fully capture the variability of these variables over time. Additionally, selection bias and recall bias must be considered, as they can affect the validity of the results. These biases should be minimized through appropriate study design and analysis. Lastly, potential sources of error with the MyotonPRO device include variability in placement, operator technique, environmental factors, and patient condition, all of which may affect measurement accuracy. These limitations should be considered when interpreting the results. Therefore, future studies should include larger sample sizes and longitudinal designs or therapies designed to enhance the robustness of the findings. They should also consider using more objective assessment tools or methods to reduce potential biases related to patient-reported outcomes. Moreover, the presence of comorbidities that may influence the study results should be carefully considered.

## 5. Conclusions

The MMPs of the PF on both sides of women with MS have lower tone and stiffness and increased elasticity and viscoelastic properties than women without MS. There were fewer differences in the MMPs of the lumbar paravertebral muscles in these women, with greater tone and stiffness on the right side in women who did not have MS. Finally, there was a moderate positive correlation between the MMPs of the PF of women with MS and clinical data related to pelvic floor dysfunction (which is not seen in healthy women) and the EDSS score, as well as a relationship between age and the MMPs of the lumbar paravertebral musculature in both groups. Thus, it is important to conduct an objective evaluation of the pelvic floor muscle properties in women with multiple sclerosis. This would enable the development of tailored preventive treatments and the ongoing assessment of their effectiveness in improving muscle function and overall quality of life.

## Figures and Tables

**Table 1:** Sociodemographic and clinical data between the groups.

Outcomes	Cases	Controls	*p*-Value
Age (years)	45.18 ± 10.69	44.73 ± 10.70	0.89
BMI (kg/m^2^)	24.86 ± 4.55	24.82 ± 4.46	0.98
PFDI-20	62.69 ± 42.95	31.58 ± 39.26	0.005
PFIQ-7	49.74 ± 48.24	21.28 ± 34.51	0.018
Number of births			0.471
0	10 (45.45%)	11 (50%)	
1	8 (36.36%)	4 (18.18%)	
2	3 (13.64%)	4 (18.18%)	
3	1 (4.55%)	3 (13.64%)	
GPAQ			0.066
Mild	6 (27.27%)	1 (4.55%)	
Moderate	9 (40.91%)	8 (36.36%)	
Intense	7 (31.82%)	13 (59.09%)	
UI type			0.346
Does not present	8 (36.36%)	10 (45.45%)	
Effort	4 (18.18%)	7 (31.82%)	
Urgency	8 (36.36%)	3 (13.64%)	
Mixed	2 (9.09%)	2 (9.09%)	

Results expressed as: mean ± standard deviation and frequency (percentage). Variables expressed as BMI: body mass index; PFDI-20: Validated Spanish version of the Pelvic Floor Distress Inventory; PFIQ-7: Validated Spanish version of the Pelvic Floor Impact Questionnaire–Short Form; GPAQ: Validated Spanish version of the Global physical activity questionnaire; UI: urinary incontinence.

**Table 2:** Comparison of the pelvic floor muscle mechanical properties between the two groups.

Outcomes	Cases (*n* = 22)	Controls (*n* = 22)	*p*-Value
FREQ-R PF (Hz)	13.02 ± 1.86	16.28 ± 1.91	<0.001
STIFFNESS-R PF (N/m)	174.77 ± 53.58	264.77 ± 60.94	<0.001
DECREM-R PF	0.93 ± 0.13	1.13 ± 0.23	0.001
RELAX-R PF (ms)	22.62 ± 4.39	16.46 ± 2.02	<0.001
CREEP-R PF (De)	1.17 ± 0.23	0.93 ± 0.08	<0.001
FREQ-L PF (Hz)	13.48 ± 2.17	16.31 ± 2.09	<0.001
STIFFNESS-L PF (N/m)	183.36 ± 64.87	260.86 ± 70.53	<0.001
DECREM-L PF	0.94 ± 0.15	1.13 ± 0.24	0.002
RELAX-L PF (ms)	21.83 ± 4.91	6.31 ± 2.19	<0.001
CREEP-L PF (De)	1.12 ± 0.19	0.91 ± 0.1	<0.001

Results expressed as the mean ± standard deviation. Variables expressed as FREQ-R: Right *Frequency*; STIFFNESS-R: Right dynamic stiffness; DECREM-R: Right *Decrement*; RELAX-R: Right *Relaxation*; CREEP-R: Right Fluency; FREQ-L: Left *Frequency*; STIFFNESS-L: Left dynamic stiffness; DECREM-L: Left *Decrement*; RELAX-L: Left *Relaxation*; CREEP-L: Left Fluency; De: Deborah number.

**Table 3:** Comparison of the muscle mechanical properties of the lumbar muscles between the two groups.

Outcomes	Cases (n = 22)	Controls (n = 22)	*p*-Value
FREQ-R Lumbar (Hz)	13.61 ± 2.06	15.76 ± 3.76	0.026
STIFFNESS-R Lumbar (N/m)	242.19 ± 53.56	304.36 ± 105.02	0.019
DECREM-R Lumbar	1.36 ± 0.25	1.47 ± 0.29	0.189
RELAX-R Lumbar (ms)	23.11 ± 4.54	19.63 ± 6.7	0.053
CREEP-R Lumbar (De)	1.39 ± 0.26	1.2 ± 0.38	0.06
FREQ-L Lumbar (Hz)	14.25 ± 1.78	16.01 ± 4.22	0.083
STIFFNESS-L Lumbar (N/m)	256.57 ± 55.38	317 ± 143.98	0.078
DECREM-L Lumbar	1.42 ± 0.31	1.44 ± 0.31	0.898
RELAX-L Lumbar (ms)	21.37 ± 3.56	19.81 ± 7.57	0.391
CREEP-L Lumbar (De)	1.3 ± 0.2	1.2 ± 0.41	0.355

Results expressed as the mean: ± standard deviation and the mean difference (95% CI). Variables expressed as FREQ-R: Right *Frequency*; STIFFNESS-R: Right dynamic stiffness; DECREM-R: Right *Decrement*; RELAX-R: Right *Relaxation*; CREEP-R: Right Fluency; FREQ-L: Left *Frequency*; STIFFNESS-L: Left dynamic stiffness; DECREM-L: Left *Decrement*; RELAX-L: Left *Relaxation*; CREEP-L: Left Fluency; De: Deborah number.

## Data Availability

The original contributions presented in this study are included in the article. Further inquiries can be directed to the corresponding author.
